# Fermented Royal Jelly Enriched With 10‐Hydroxydecanoic Acid and Its Potential for Enhancing Mucosal Immunity

**DOI:** 10.1002/fsn3.70041

**Published:** 2025-02-18

**Authors:** Hayate Itatani, Ayanori Yamaki, Kaori Konishi, Hideto Okamoto, Nobuaki Okumura, Norihiro Shigematsu, Shogo Misumi, Shinji Takenaka

**Affiliations:** ^1^ Institute for Bee Products & Health Science, R&D Department Yamada Bee Company, Inc. Okayama Japan; ^2^ Environmental Microbiology, Division of Agrobioscience, Graduate School of Agricultural Science Kobe University Kobe Japan; ^3^ Yamada Bee Company Group Institute for Beauty Science, R&D Department Yamada Bee Company, Inc. Tokyo Japan; ^4^ R&D Department Yamada Bee Company, Inc. Okayama Japan; ^5^ Department of Environmental and Molecular Health Sciences, Faculty of Medical and Pharmaceutical Sciences Kumamoto University Kumamoto Japan

**Keywords:** 10‐hydroxydecanoic acid, intestinal immunity, *Lactobacillus panisapium*, M cell differentiation, royal jelly

## Abstract

Royal jelly (RJ) is known to contain 10‐hydroxydecanoic acid (10HDAA), which has been shown to have immune activation properties, including the promotion of M cell differentiation. However, the natural concentration of 10HDAA in RJ is relatively low. To enhance the functional use of RJ as an immunostimulatory food ingredient, this study aimed to increase its 10HDAA content using bacteria capable of converting 10‐hydroxy‐2‐decenoic acid (10H2DA) to 10HDAA in RJ. A lactic acid bacterium, *Lactobacillus panisapium*, was isolated from the digestive tract of queen bees and demonstrated a high capacity to convert 10H2DA to 10HDAA. Using the isolated strain, fermented RJ (fRJ) with a fivefold increase in 10HDAA content was produced compared to raw RJ. Preliminary evaluations of fRJ's immune‐stimulating effects revealed significant benefits, including enhanced M cell differentiation, activation of macrophage phagocytic ability, and increased immunoglobulin (Ig) A secretion in individuals with reduced salivary IgA levels. Safety assessments confirmed that fRJ is safe for consumption. In summary, fRJ enriched with 10HDAA was produced and demonstrated potential as an immune‐stimulating food.

## Graphical abstract

We isolated the *Lactobacillus panisapium* strain M1 from queen bee digestive tracts, capable of converting 10‐hydroxy‐2‐decenoic acid (10H2DA) in RJ to 10‐hydroxydecanoic acid (10HDAA). Through cultivation in protease‐treated RJ broth, we successfully produced fermented RJ (fRJ) containing significantly elevated 10HDAA levels compared to traditional RJ.

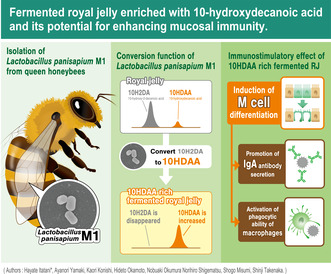

## 1 Introduction

Royal jelly (RJ) is a nutrient‐rich secretion produced by honeybees from pollen, nectar, and honey, which they digest and secrete through their hypopharyngeal and mandibular glands. Various bioactivities have already been reported for RJ, and it is known for its antioxidant, antitumor, anti‐aging, neurostimulatory, anti‐inflammatory, and anti‐aging effects (Kunugi and Mohammed Ali 2019; Pasupuleti et al. 2017). Moreover, enzymatically treated RJ products have demonstrated additional bioactivities. For instance, protease‐treated RJ (pRJ) and its ether extracts have been shown to enhance antigen‐specific immunoglobulin (Ig) A responses and increase the expression of the universal M cell marker glycoprotein 2 (gp2), respectively (Kai et al. 2013).

Among the various bioactive compounds in raw RJ, two significant fatty acids stand out: trans‐10‐hydroxy‐2‐decenoic acid (10H2DA) and 10‐hydroxydecanoic acid (10HDAA) (Yu et al. 2023). Among them, 10H2DA is the predominant fatty acid in raw RJ, present at approximately 1.2%–4.5%, while 10HDAA is found at lower concentrations, around 0.3%–1.2% (Yu et al. 2023). Despite their structural similarities, 10H2DA and 10HDAA exhibit distinct biological activities.

Previous research has shown that 10H2DA contributes to the longevity and reproductive capacity of queen bees, and it plays a crucial role in larval development (Kunugi and Mohammed Ali 2019). It also regulates glycerol transport in the liver by downregulating aquaporin 9 (AQP9) gene expression through the activation of Akt and AMP‐activated protein kinase (AMPK) phosphorylation (Usui et al. 2019). In humans, 10H2DA demonstrates estrogen‐like and filaggrin‐producing effects, contributing to skin health and hormonal balance (Mishima et al. 2005; Maeda et al. 2022). Additionally, the bioconversion of 10HDAA to 10H2DA via one‐step whole‐cell catalysis has been established for industrialization (Fang et al. 2024).

In contrast, 10HDAA is known for its potent anti‐inflammatory properties (Takahashi et al. 2013) and its ability to stimulate the immune system by increasing the number of M cells in intestinal Peyer's patches, which enhances antigen‐specific IgA expression—an effect not seen with 10H2DA (Isayama et al. 2020). Although 10H2DA and 10HDAA are the major fatty acids in raw RJ, 10HDAA has a stronger capacity to stimulate mucosal immunity.

However, the naturally low concentration of 10HDAA in raw RJ limits its potential for enhancing immunity. To address this, we hypothesized that converting 10H2DA into 10HDAA would amplify RJ's effects on mucosal immunity. Although there are no existing reports on bioconversion through whole cells or enzymatic processes for this purpose, bioconversion presents a promising approach. Specifically, microbial enzymes, such as those from *Clostridium sporogenes* , which reduce carbon–carbon double bonds in similar substrates like cinnamic acid (Mordaka et al. 2018), could be harnessed for this process.

The aim of this study was to isolate a bacterium from the honeybee gut microflora capable of converting 10H2DA to 10HDAA and to produce RJ enriched with 10HDAA using the isolate. Additionally, the immunostimulatory potential of the bio‐treated RJ was evaluated, focusing on its ability to enhance mucosal immunity by promoting M cell differentiation, activating macrophages, and increasing IgA secretion.

## 2 Materials and Methods

### 2.1 Chemicals and Cultivation Media

Pepsin and protease YBFII were purchased from FUJIFILM Wako Pure Chemical Corp. (Osaka, Japan) and Yamada Bee Company Inc. (Okayama, Japan), respectively. The raw RJ standardized to contain 1.6% 10H2DA and 0.4% 10HDAA was obtained from Yamada Bee Company Inc. All other chemicals, including 10H2DA and 10HDAA, were of analytical grade. De Man–Rogosa–Sharpe (MRS) broth (Becton, Dickinson, and Corp., Franklin Lakes, NJ, USA) and transgalactosylated oligosaccharide (TOS) propionate (Yakult Pharmaceutical Industry Co. Ltd., Tokyo, Japan) were used for the isolation, cultivation, and enrichment of *Lactobacillus* species and *Bifidobacteria*, respectively. An anaerobic jar and anaerobic gas‐producing pouch (Anaeropack Kenki, Mitsubishi Gas Chemical Co. Inc., Tokyo, Japan) were used for anaerobic cultivation. Dulbecco's Modified Eagle's Medium (D‐MEM) (FUJIFILM Wako Pure Chemical Corp.) and fetal bovine serum from HyClone Laboratories Inc. were used to cultivate the cells of the macrophage‐like strain J774.1. Fluoresbrite polychromatic red microspheres (Polysciences Inc., Warrington, PA, USA) were used to assess macrophage phagocytic capacity.

### 2.2 Isolation of 10H2DA‐Converting Bacteria

The final goal of this study was to develop bio‐treated RJ enriched with 10HDAA for use as an immunostimulatory food. Therefore, we focused on isolating *Lactobacillus* and *Bifidobacterium* species, which are commonly used in food products. Given that RJ is an essential secretion for the nutrition of honeybee larvae and adult queens, previous studies have isolated *Lactobacillus* species from the honey stomach, gut, and bee bread of honeybees (Wang et al. 2018). Consequently, these honeybee components were selected as sources for bacterial isolation.

#### 2.2.1 Preparation of Digestive Tract Samples From Honeybees and Honeybee Larvae

Digestive tracts from workers and queen bees, along with whole bee larvae, were obtained from the Yamada Bee Company. For adult bees, 1 g of the gastrointestinal tract was aseptically mixed with 9 mL of sodium–potassium phosphate buffer (pH 6.0) in a 15 mL test tube. The mixed solutions were designated as digestive tract samples. For larvae, an equivalent amount of tissue was prepared in the same manner.

#### 2.2.2 Separation of Lactic Acid Bacteria and Bifidobacteria

The prepared samples from larvae, workers, and queen bees were diluted 10–100,000 times with sodium–potassium phosphate buffer (pH 6.0) and spread onto MRS and TOS propionate agar plates. Plates were incubated for 96 h at 35°C under anaerobic conditions using AnaeroPac Kenki, an oxygen absorber and carbon dioxide generator. Colonies that grew well on the plates were inoculated into MRS liquid medium and incubated anaerobically, as described above. For initial separation and characterization, isolated samples were tested for Gram stain and catalase activity. Single‐colony isolation was carried out using TOS agar plates (once) at 37°C for 72 h and MRS agar plates (repeated three times) at 35°C for 96 h.

#### 2.2.3 Selection of Strains Capable of Converting 10H2DA to 10HDAA

To select a strain capable of converting 10H2DA to 10HDAA (referred to as the 10H2DA‐converting strain), isolates were anaerobically cultivated in MRS liquid medium containing 0.4% (w/v) 10H2DA at 35°C for 96 h. After centrifugation, the culture supernatants were analyzed by HPLC for conversion. The concentrations of 10H2DA and 10HDAA were measured using a Shimadzu prominence HPLC system (SHIMADZU Co., Kyoto, Japan) with a Sunniest C18 column (4.6 mm i.d. × 250 mm; ChromaNik Technologies Inc., Osaka, Japan) at a flow rate of 1 mL/min at 40°C. 10H2DA and 10HDAA were eluted with 0.1% TFA‐acetonitrile (75:25, v/v) and detected by photodiode array detection and evaporative light scattering detection, respectively. The retention times of 10H2DA and 10HDAA were 10.51 and 13.47 min, respectively (Figure S1a). Finally, five 10H2DA‐converting strains were selected.

### 2.3 Identification of 10H2DA‐Converting Strain

#### 2.3.1 MALDI/TOF MS and 16S rRNA Gene Analyses

The protein mass spectra of the test bacteria obtained using matrix‐assisted laser desorption ionization time‐of‐flight mass spectrometry (MALDI‐TOF MS) were compared with those of bacterial species data in the library at the Central Institute for Experimental Medicine and Life Science (Kanagawa, Japan). In addition, 16S rDNA sequencing, molecular phylogenetic analysis, genomic analysis, and average nucleotide identity testing were performed at Techno Suruga Laboratory Co. Ltd. (Shizuoka, Japan). Comparative analysis was performed for *L. panisapium* and *L. bombicola*, which are closely related, as described below.

#### 2.3.2 Major Fatty Acid Analysis

The cellular fatty acid profile of *L. panisapium* comprised C16:0 (content %, 16.2), C18:1ω9c (54.8%), and iso‐C19:0 (10.5%) as the major fatty acids (Wang et al. 2018). The profile is one of the properties used to classify the species and closely related species, such as *L. bombicola* (15.2%, 69.0%, and 2.1%), *L. apis* (13.0%, 64.6%, and 7.0%), and *L. helsingborgensis* (18.6%, 63.0%, and not detected) (Wang et al. 2018). Among these four species, *L. panisapium* has a relatively high content of iso‐C19:0. The preparation of cultivated cells, freeze‐drying, and esterification of fatty acids were carried out according to previously reported procedures (Ikemoto et al. 1978). The cellular fatty acid methyl esters were dried, and the free fatty acids were esterified with a hydrogen chloride–methanol reagent (5%–10%) for 3 h at 100°C in a screw‐cap tube. Methyl lauric acid (C12:0) was used as an internal standard. The esterified fatty acids were analyzed using a GC‐MS‐QP2010 Ultra apparatus (SHIMADZU Co., Kyoto, Japan) with a fused silica capillary column (InertCap 1MS; 0.25 mm × 30 m; GL Science, Osaka, Japan). Helium was the carrier gas at a linear velocity of 35 cm/s. The column was held at 150°C for 2 min and then heated stepwise from 150°C–280°C at 4°C/min. The MS interphase and ion source temperatures were 200°C and 250°C, respectively.

#### 2.3.3 Utilization of Sugars

Melibiose, D‐sorbitol, sucrose, maltose, raffinose, and rhamnose were added to tubes of peptone yeast extract (PY) medium as filter‐sterilized solutions at a final concentration of 20.0 g/L. Growth was determined using the method described by Ma et al. (2017).

### 2.4 Survival Rates Under Simulated Stomach and Gastrointestinal Conditions

The 10H2DA‐converting strain, after anaerobic cultivation in MRS medium at 35°C for 96 h, was washed with 0.5% (w/v) NaCl solution. The bacterial cells were resuspended in NaCl solution to a final concentration of 5%. Pepsin was dissolved in this solution to achieve a concentration of 0.3% (w/v), and the pH was adjusted to 3.0 using 1 N HCl. This bacterial suspension was then mixed with 1 mL of pepsin solution and 0.5 mL of saline solution and preincubated at 37°C. Subsequently, 100 μL of the bacterial suspension was combined with 500 μL of pepsin solution and 150 μL of NaCl solution, and the mixture was preincubated at 37°C for 1 h. A 2 μL sample of this mixture was spread onto MRS agar plates and incubated at 35°C for 96 h under anaerobic conditions.

Artificial bile acids were used to evaluate bile acid resistance. MRS medium was prepared with 0.3% Oxgall by dissolving 60 mg of Oxgall in 20 mL of MRS medium. The bacteria grown in MRS medium were seeded in 96‐well microplates and centrifuged (3000 × *g*, 4°C, 10 min) to collect bacterial precipitates. These precipitates were then inoculated into MRS medium alone or MRS medium containing Oxgall and incubated under anaerobic conditions for 6 h, and the absorbance (OD 660) of each well was measured.

### 2.5 Bioconversion of 10H2DA to 10HDAA in RJ Solution

The bioconversion of 10H2DA to 10HDAA was investigated in both the untreated and pRJ solutions. In this study, the conversion of 10H2DA by the isolated strain and its product were referred to as RJ fermentation and fermented RJ (fRJ), respectively. Raw RJ (untreated raw RJ, nRJ) was subjected to enzymatic degradation to produce pRJ. For pRJ preparation, water was added to nRJ to achieve a 50% solution, which was then adjusted to pH 7.8 with NaOH. Protease YBFII was added to a final concentration of 0.5% (w/v), and the mixture was digested at 50°C for 2 h. Both nRJ and pRJ were diluted fivefold with purified water to prepare the fermentation mother liquor, which was adjusted to pH 6.5 using 2 N NaOH and aerated with nitrogen gas to achieve a dissolved oxygen level of zero. The 10H2DA‐converting strain, strain M1, was cultured in MRS medium, and this preculture solution was added to the mother liquor at a concentration of 10% of the total volume. The bioconversion process was performed at 35°C for 120 h using a Bioneer‐Neo tabletop incubator (Marubishi Bioengineering Co. Ltd., Tokyo, Japan), and pH and dissolved oxygen were controlled by a bioprocess controller. Nitrogen gas was bubbled through the solution for 30 min daily. The fermented solution was sterilized by heating at 80°C for 20 min. The concentrations of 10H2DA and 10HDAA were quantified using HPLC, as described above (see Section 2.2.3).

### 2.6 Safety Analysis of fRJ

The bioconversion in pRJ solution was much higher than that in nRJ, as described below (see Section 3.3). The fermented pRJ solution was collected, frozen, and dried using a freeze dryer for the preparation of fRJ powder. Its tablet or capsule was also used for safety analysis, M cell differentiation, macrophage phagocytosis, and salivary IgA, as described below. In vitro, animal, and human safety studies were conducted on fRJ powder (lot: YHRP‐M‐201022–2). The powder was obtained by adding 25.8% excipient to the solid content of the fRJ solution, followed by lyophilization. The studies were conducted by the BioSafety Research Center Inc. (Shizuoka, Japan), and human overdosage studies were conducted by the Research Center for Immunological Analysis Inc. (Okayama, Japan). In vitro reverse mutation studies were performed using bacteria and acute and repeated‐dose studies in animals. This study was approved by the Ethical Review Board of Yamada Bee Company Inc. (approval no. 16000050).

### 2.7 Evaluation of M Cell Differentiation‐Promoting Activity

The ability of fRJ to promote M cell differentiation, as previously reported for 10HDAA, was evaluated. Using the method described by Isayama et al. (2020), monolayer‐treated Caco‐2 cells were co‐cultured with fRJ powder (1.7 mg/well), and the expression levels of glycoprotein 2 (GP2), a marker of mature M cells, were analyzed using fluorescence‐activated cell sorting. As a control, pRJ (without bio‐treatment) was used instead of fRJ.

### 2.8 Measurement of Macrophage Phagocytosis

The phagocytic ability of fRJ was assessed using cells of the macrophage‐like strain J774.1. The J774.1 cells were cultured in D‐MEM containing 10% fetal bovine serum (HyClone Laboratories Inc., Logan, UT, USA) and 1% penicillin–streptomycin–glutamine (Thermo Fisher Scientific K.K., Tokyo, Japan). The fRJ (lot: 20200512) was added to the culture medium at a concentration of 1 mg/mL, and the solution was heated at 37°C for 15 min and sterile filtered through a 0.20 μM filter.

Two milliliters of Fluoresbrite polychromatic red microspheres (Polysciences Inc., Warrington, PA, USA.) (5.68 × 10^9^ beads/mL) were measured and centrifuged at 2000 × *g* for 5 min, and the supernatant was separated. Two milliliters of culture medium were added to the precipitated beads to obtain a suspension of 4 × 10^8^ beads/mL.

The J774.1 cells were harvested every 3–4 days at 1–2 × 10^5^ cells/mL and used for testing. Cells were seeded at 2 × 10^5^ cells/well in 48‐well plates and incubated for approximately 16 h at 37°C in a 5% CO_2_ incubator. After preculture, the culture medium was removed, and fRJ prepared to 1 mg/mL in culture medium was added and incubated for 1 h at 37°C in a 5% CO_2_ incubator. Phycoerythrin‐labeled polystyrene bead suspension was added to reach 4 × 10^6^ beads/well and incubated in a 5% CO_2_ incubator at 37°C for 2.5 h. After incubation, the cells were collected, and the phagocytosis rate and mean fluorescence intensity (MFI) were measured using a flow cytometer.

### 2.9 Effect on Salivary Ig A in Two Clinical Studies

The effect of orally administered fRJ on salivary IgA levels was tested in healthy adults. The first study was approved by the Ethical Review Board of Yamada Bee Company Inc., on January 11, 2021 (approval no. 2020027) and was conducted in accordance with the principles of the Declaration of Helsinki. All participants provided written informed consent, and 12 participants with low IgA secretion rates were selected for the study. This study was an open‐label study, and the test food was fRJ containing 20 mg 10HDAA per three tablets as a standard. Participants were administered three tablets of the test sample once a day for 3 weeks. The primary endpoint was the rate of salivary IgA secretion (g/min), and the secondary endpoints were salivary IgA concentration (g/mL), visual analog scale (VAS) questionnaire (sleep quality, fatigue, and stress levels), bowel frequency, and cold symptoms. Tests were performed before ingestion and at 1, 3, 7, 14, 21, and 35 days after ingestion (2 weeks after the end of ingestion), and each item was scored. Measurements are presented as mean ± standard deviation, and comparisons were made using a paired *t*‐test.

Additionally, the effect on human salivary IgA was tested using a selection procedure that did not restrict participants to those with a low IgA secretion rate. The second study was conducted with the approval of the Ethical Review Board of Yamada Bee Company Inc. on July 27, 2021 (approval no. 2021007). This study was conducted in accordance with the principles of the Declaration of Helsinki. All participants provided written informed consent, and 20 were selected for the study. This open‐label study investigated the effect of fRJ on salivary IgA in healthy adults. The test diet consisted of fRJ (13 capsules per day) containing at least 20 mg of 10HDAA. Twenty healthy Japanese adults (men and women) aged 18 years or older consumed the fRJ‐containing diet for 8 weeks, and the rate of salivary IgA secretion (g/min) was assessed.

### 2.10 Statistical Analysis

Means and standard deviations were calculated using basic statistics. JMP 5.1 (SAS Institute Japan Ltd., Tokyo, Japan) was used for statistical analyses of clinical studies, with a two‐sided significance level of 5% for all tests. The calculated data are presented in tables and graphs as the mean ± standard deviation. The phagocytosis rate and MFI were compared between the control and fRJ groups using Dunnett's test. GraphPad Prism version 7 (GraphPad Software, Boston, MA, USA) was used for statistical analyses of in vitro studies.

## 3 Results

### 3.1 Isolation of Lactic Acid Bacteria and Bifidobacteria Capable of Converting 10 H2DA to 10 HDAA From Honeybees

A total of 4, 73, and 80 Gram‐positive, catalase‐negative strains were isolated from larvae, workers, and queen honeybees, respectively. These strains were cultured in an MRS medium containing 10H2DA, and the conversion of 10H2DA to 10HDAA in the culture was measured. Out of the 157 strains tested, 5 strains (QB 3‐1‐4, QB 3‐1‐9, QB 3‐1‐10, QB 3‐2‐4, and QB 9‐1‐6) demonstrated the ability to convert 10H2DA to 10HDAA. Notably, strain QB 9‐1‐6 (referred to as strain M1) exhibited an approximately 100% conversion rate. The other four strains showed < 50% conversion efficiency, with more than 50% of 10H2DA remaining in the culture supernatant. Therefore, strain M1 was selected for further analysis.

### 3.2 Identification of 10H2DA‐Converting Lactic Acid Bacteria

**FIGURE 1:**
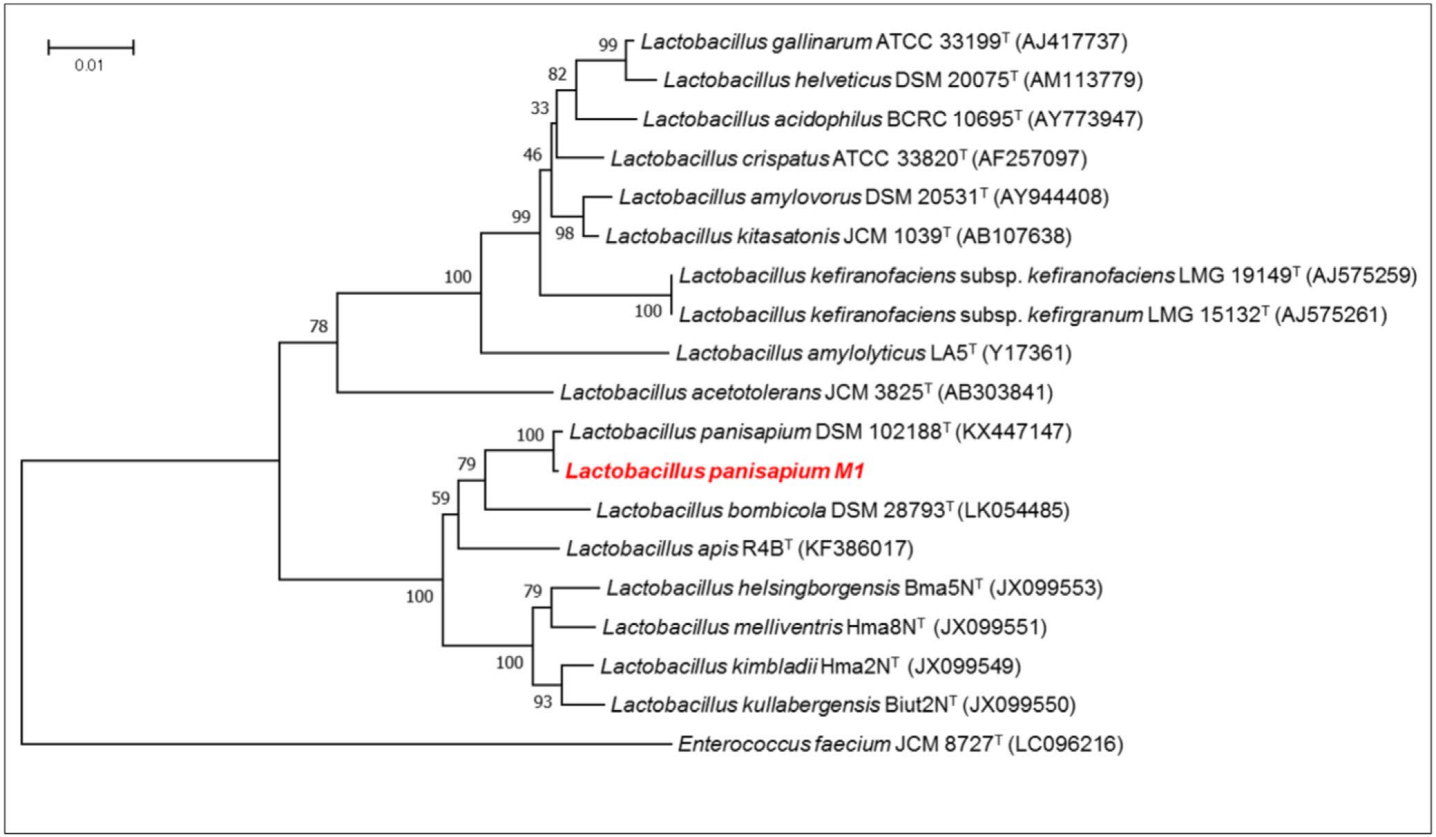
Neighbor‐joining phylogenetic tree based on 16S rRNA gene sequences showing the relationship between strain M1 and its phylogenetically close relatives. Bootstrap values based on 1000 replications are listed as percentages at the branching points.

We attempted to identify and classify the five strains based on bacterial protein spectra using MALDI/TOF MS and 16S rRNA gene sequencing. However, their mass spectral data did not match any of the tested bacterial protein spectra in the Bruker Daltonics MALDI Biotyper libraries (MBT Compass Library V12 and MBT Filamentous Fungi Library V5), making identification impossible. The 16S rRNA gene sequences of the five strains, including strain M1, were 100% identical. A homology search revealed that the strains had the highest identity (99.7%) with *L. panisapium* Bb2‐3 (KX447147). Among the five isolates, strain QB9‐1‐6 was selected based on its 10H2DA conversion ability and was designated as strain M1. Phylogenetic analysis indicated that strain M1 belongs to a group consisting of *L. panisapium* and its closely related species, *L. bombicola*, *L. apis*, and *L. helsingborgensis* (Wang et al. 2018; Figure 1). These related species can be classified according to their sugar assimilation ability and cellular fatty acid content (Wang et al. 2018). The cellular fatty acid profile of strain M1 comprised C18:1ω9c (10%), C16:0 (20%), and iso‐C19:0 (30%) as the major fatty acids (> 10%). This profile differed from those of closely related species (Table 1). Sugar assimilation performance was found to be facultative toward D‐glucose, D‐fructose, D‐mannose, *N*‐acetylglucosamine, amygdalin, esculin, iron citrate, salicin, and D‐cellobiose. Compared to previous findings on sugar assimilation in closely related species, strain M1 assimilated melibiose and sucrose but not maltose and D‐sorbitol. These findings indicate that strain M1 belongs to the species *L. panisapium*. Additionally, strain M1 showed resistance to gastric and bile acids.

**TABLE 1:** Comparison of cellular fatty acid content (%s) of strain M1 with its phylogenetically closest relatives.

Fatty acid	1	2	3	4	5
Saturated					
C_14:0_	2.53 ± 0.24	1.3	0.8	0.6	0.9
C_15:0_	0.202 ± 0.11				
C_16:0_	20.632 ± 1.07	16.2	15.2	13.0	18.6
C_18:0_	5.044 ± 0.44	5.1	3.3	5.5	6.0
Unsaturated					
C_18:1 n9_	52.318 ± 0.70	54.8	69.0	64.6	63.0
C_18:1 n7_	7.65 ± 0.39	4.9	3.9	5.4	—
C_19:1_	5.73 ± 0.31				
Branched‐chain					
C_19:0_ iso	5.26 ± 0.44	10.5	2.1	7.0	

*Note:* Strains: 1, strain M1; 2, *L. panisapium* strain Bb 2–3^T^; 3, *L. bombicola* DSM 28793^T^; 4, *L. apis* LMG 26964^T^; 5, *L. helsingborgensis* DSM 26265^T^ Wang et al. (2018).

### 3.3 Bioconversion of 10H2DA to 10HDAA in nRJ and pRJ by Strain M1

When a diluted sample of nRJ was used as the fermentation broth, the growth of strain M1 was poor, and the conversion reaction was not efficient (Figure S1). This inefficiency may be attributed to the presence of proteins like major RJ protein 1 (MRJP1), which has potential bactericidal activity (Brudzynski and Sjaarda 2015). Another possible explanation is that the proteins in nRJ are not readily usable by strain M1 but require enzymatic degradation to polypeptides, as seen in pRJ. During incubation, the amount of 10H2DA in the culture supernatant gradually decreased, and complete conversion to 10HDAA was achieved by day 5. The culturing was carried out under anaerobic conditions, as strain M1 did not grow or convert 10H2DA efficiently in the presence of oxygen (Figure S1). This result is consistent with the fact that strain M1 was isolated from the queen bee's digestive tract, which is an anaerobic environment. For practical applications, it was determined that the conversion process for RJ must be conducted under anaerobic conditions. RJ's protein, lipid, and total sugar content have been reported to be 12%–15%, 3%–6%, and 10%–16%, respectively (Chen and Chen 1995). Given the high conversion efficiency observed in the pRJ broth, it is likely that strain M1 could not efficiently utilize untreated RJ proteins as sources of carbon, nitrogen, or energy.

### 3.4 fRJ is More Potent Than pRJ in Promoting M Cell Differentiation

**FIGURE 2:**
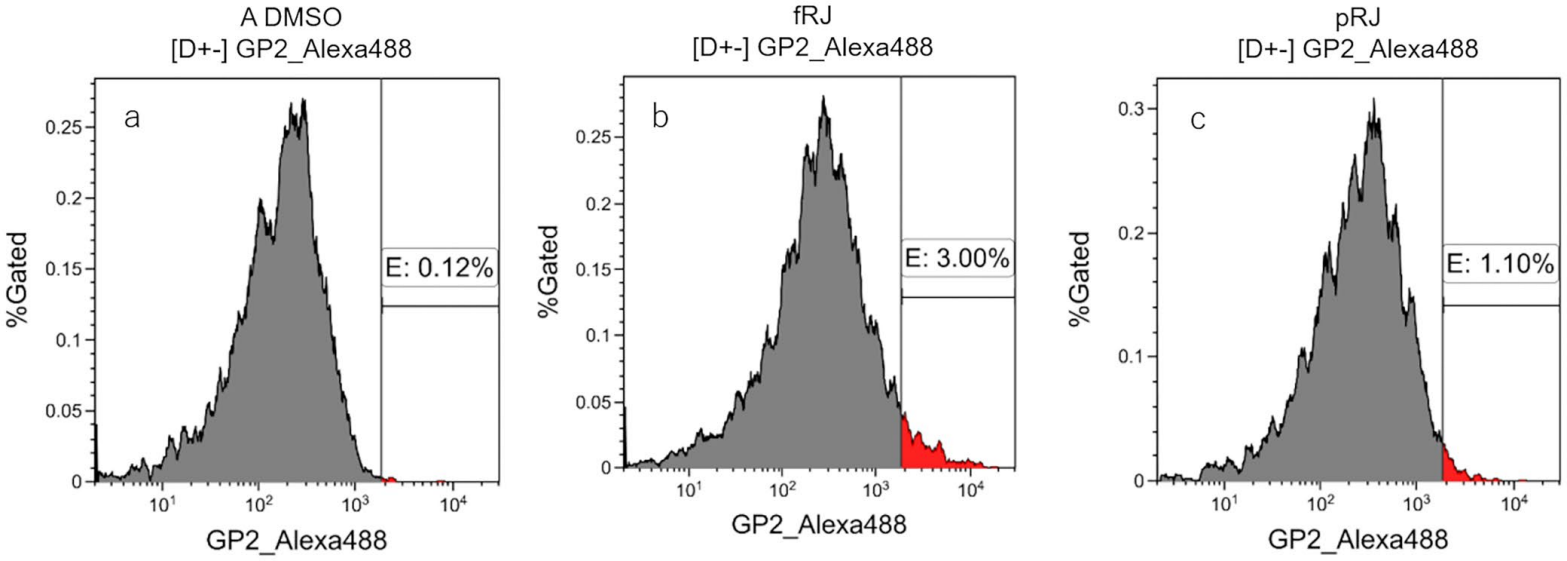
Assessment of M cell differentiation‐promoting activity of RJ. Representative flow cytometry results showing GP2 expression in Caco‐2 cells treated with 0.85 mg/mL fRJ and pRJ. Comparison of the occupancy of GP2 high‐expressing areas (red). fRJ, the protease‐treated royal jelly was fermented by strain M1; pRJ, raw royal jelly was subjected to protease degradation.

The M cell differentiation‐promoting effects of pRJ and fRJ were assessed using equal amounts of pRJ and fRJ. pRJ showed higher GP2 expression than DMSO, while fRJ showed the highest expression (Figure 2). This suggests that fRJ has a more potent effect in promoting M cell differentiation per unit weight, potentially reducing the required intake of RJ.

### 3.5 Safety Analysis of Fermented RJ as a Food Supplement

#### 3.5.1 Reverse Mutation Test Using Bacteria

The number of reverse mutant colonies in the fRJ‐treated group was no more than twice that in the negative control group for either the −S9‐ or +S9‐treated strains. No growth‐inhibitory effects were observed on the test strains at either dose. The positive control induced a significant reversion of the mutation in each test strain.

#### 3.5.2 Acute Toxicity Test

No animals from either sex died during the observation period. No abnormalities in general condition or weight changes were observed in any treatment group. In males, cysts and depressed foci in the kidneys were observed in the control group, diverticula in the small intestine (ileum) in the 10HDAA 51.9 mg/kg group, and transhepatic nodules in the liver in the 103.8 mg/kg group. These findings were considered spontaneous changes commonly observed. No abnormalities were observed in females. There were no deaths in either sex across the 26.0, 51.9, or 103.8 mg/kg dose groups of 10HDAA. No changes in general condition, body weight, or necropsy findings could be attributed to the administration of the test substance.

#### 3.5.3 Repeated Dose Test

None of the animals died during the experiments. Differences in body weight or body weight gain were not significantly different between the control and test groups in either sex during the treatment period. Urinalysis showed higher total urinary sodium excretion in males in the 51.9 mg/kg/day group of 10HDAA and in females in the 26.0 and 51.9 mg/kg/day groups than in the other groups. However, no change in blood sodium concentration was observed, which was presumably a result of the excretion of the sodium contained in the test substance (approximately 7.5%) and was not considered a toxic effect. The amount of positive ketone bodies and urine protein in males in the 13.0 mg/kg/day and higher dose groups increased dose‐dependently. In females, the amount of positive ketone bodies in the 26.0 and 51.9 mg/kg/day groups and urine protein in the 51.9 mg/kg/day group increased dose‐dependently. Despite these increases, toxic effects related to increased ketone and urine protein levels were not considered to be present, as blood biochemical tests showed no evidence of abnormal glucose metabolism, starvation, or renal damage, and histopathological tests showed no evidence of liver, pancreas, or kidney damage. No other abnormalities that could be attributed to the administration of the test substance were found in the general condition, body weight, food consumption, hematology, blood biochemistry, ophthalmology, organ weight, necropsy, or histopathology.

#### 3.5.4 Human Overdose Test

Twenty patients consented to participate in the screening, and 12 of the enrolled participants consumed the study diet, among which 10 participants completed the study, and two participants withdrew from the study for personal reasons. One of the 10 participants (ID: 4) did not participate in V1 due to fever but was included in the final case count due to their participation in V0 and V2. The analyses included 3 males and 7 females with a mean age of 40.1 ± 4.5 years and a mean body mass index of 21.8 ± 1.2 for males and 20.5 ± 2.3 for females. Adverse events were graded by the investigators on the following four‐point scale: “relevant,” “probably relevant,” “probably not relevant,” and “not relevant.” The number (incidence) of adverse events was 6/12 (50.0%), of which 1/12 (8.3%) was confirmed as an adverse event (rash), for which a causal relationship could not be excluded. The participant developed a “rash” during consumption of the test food (day 17) and took their anti‐allergic medication, but the symptoms did not subside and the rash spread all over the body; hence, this participant consulted a dermatologist on day 24 of consumption. The dermatologist suggested no connection to the test food but could not completely rule it out. The participant was treated with a steroid/antihistamine combination, and the symptoms abated but continued 2 weeks poststudy. The timing and duration of symptoms were not related to the duration of test food consumption, suggesting that a causal relationship was unlikely.

No serious adverse events or reactions leading to death were observed. Adverse events or reactions leading to discontinuation of the study were not observed. No clinically relevant findings were observed during physical examination, physiological examination, urinalysis, hematology, or blood biochemistry. In summary, no adverse events were clearly causally related to the test product, and no adverse events of moderate or greater severity or clinically concerning changes in laboratory values were observed. These findings suggest that it is safe for healthy adults to consume excessive amounts (five times the dose) of fRJ‐containing foods.

### 3.6 Functional Assessment of fRJ (Macrophages, IgA)

Based on the M cell differentiation‐promoting effect and safety assessment tests of fRJ, a preliminary functional assessment was performed on macrophages and IgA, which are the downstream immune cells of M cells in the intestinal immune system.

#### 3.6.1 Macrophage Phagocytosis Activity

**FIGURE 3:**
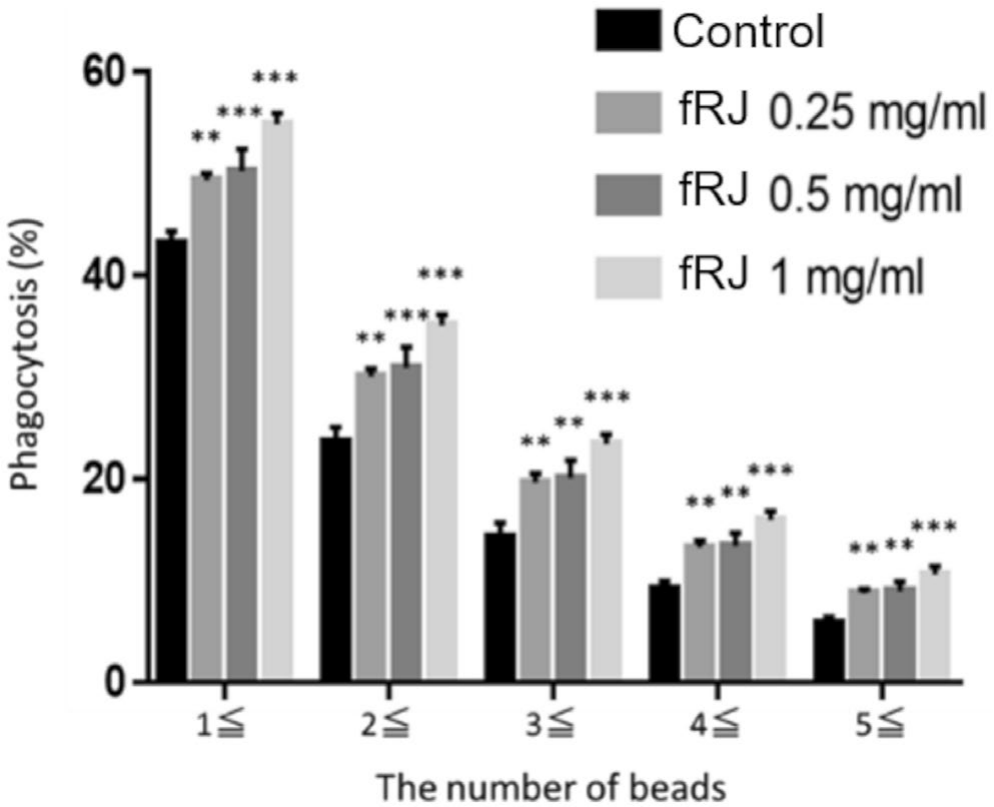
Phagocytosis rate by fRJ. Values are presented as mean ± SD. *p < 0.05, **p < 0.01, and ***p < 0.001 relative to the control using Dunnett's test. fRJ, the protease‐treated royal jelly was fermented by strain M1.

**FIGURE 4:**
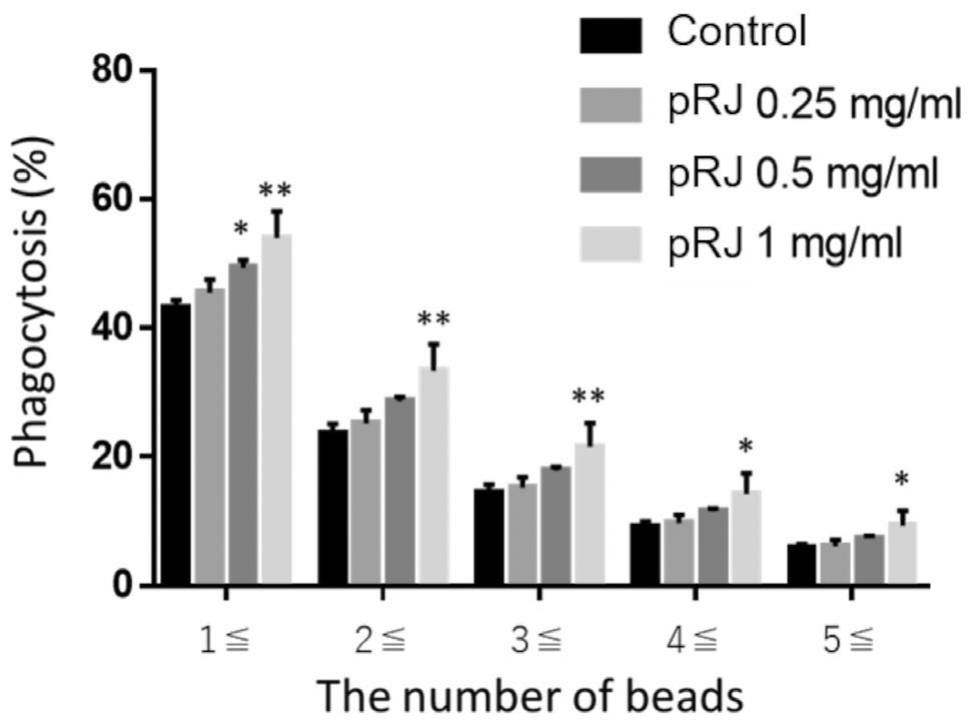
Phagocytosis rate by pRJ. Values are presented as mean ± SD. *p < 0.05, **p < 0.01, and ***p < 0.001 relative to the control using Dunnett's test. pRJ, protease‐treated royal jelly.

**FIGURE 5:**
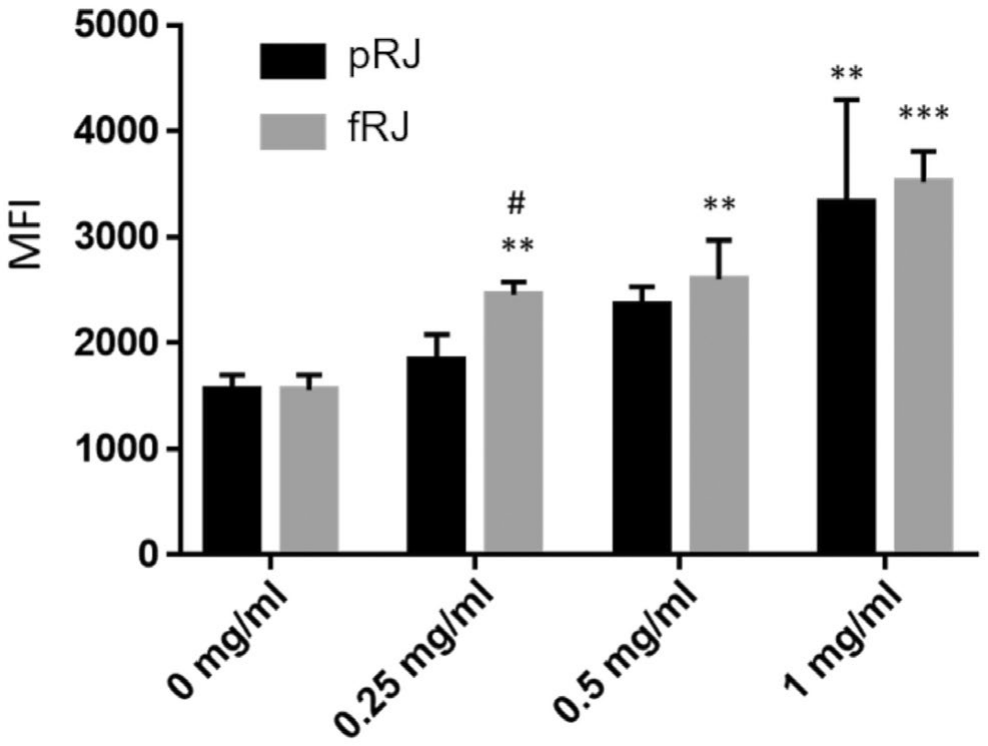
Average fluorescence intensities using fRJ and pRJ. Values are presented as mean ± SD. *p < 0.05, **p < 0.01, and ***p < 0.001 relative to the control using Dunnett's test. fRJ, the protease‐treated royal jelly was fermented by strain M1; MFI, mean fluorescence intensity; pRJ, raw royal jelly was subjected to protease degradation.

Compared to the control, fRJ significantly increased the phagocytic rate and MFI at all tested volumes (Figures 3 and 4). The MFI was significantly higher in the fRJ‐treated group than in the pRJ group at 0.25 mg/mL (Figure 5). The phagocytosis rate and MFI were compared between the control and fRJ groups using Dunnett's test. GraphPad Prism version 7 (GraphPad Software, Boston, MA, USA) was used for statistical analyses of in vitro studies.

#### 3.6.2 Salivary IgA Secretion Rate

**FIGURE 6:**
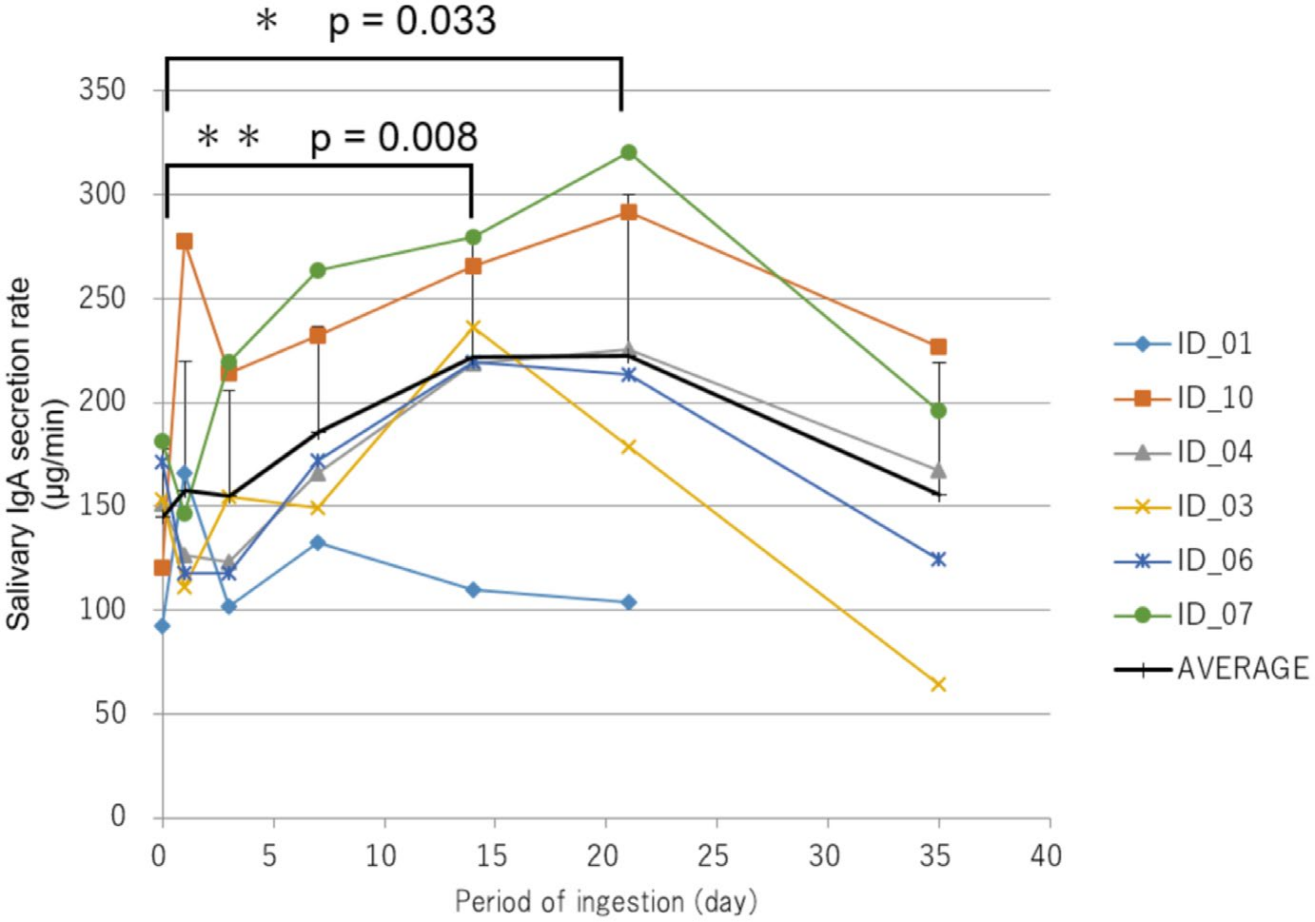
IgA secretion rate (μg/min) in six subjects with a low IgA secretion rate before starting treatment.

Twelve participants (four men and eight women) completed the study, with a mean age of 38.3 ± 15.2 years. After 1, 3, 7, 14, 21, and 35 days of ingestion, salivary IgA secretion rates were not significantly different from preingestion levels (Figure S2). However, participants with low salivary IgA secretion rates at preingestion showed a significant increase on days 14 and 21 compared to preingestion (Figure 6). The number of bowel movements per day was averaged per week, and no significant differences were observed between the second and third weeks of ingestion compared to the first week. Seven examinations and a cold symptom questionnaire were conducted, but the low incidence of symptoms (three cases) made it unfeasible to evaluate these results relative to preingestion. A significant increase in salivary IgA secretion rates was also observed after 8 weeks of ingestion in studies where participants were not limited to those with potentially low IgA secretion rates (Table S1). Means and standard deviations were calculated using basic statistics. JMP 5.1 (SAS Institute Japan Ltd., Tokyo, Japan) was used for statistical analyses of clinical studies, with a two‐sided significance level of 5% for all tests. The calculated data are presented in tables and graphs as the mean ± standard deviation.

## 4 Discussion

In this study, we successfully isolated a lactic acid bacterium, strain M1, capable of converting 10H2DA to 10HDAA and identified it as *L. panisapium*. The low amount of 10HDAA in raw RJ was a major issue, but during the RJ fermentation process, strain M1 converted 10H2DA to 10HDAA, and the amount of 10HDAA increased approximately fivefold higher than that of the original RJ broth. Based on the immunostimulatory effect and safety analyses of the fRJ solution during ingestion, RJ could be used as food by increasing the 10HDAA content.

Several lactic acid bacteria have been isolated from the stomach (Olofsson et al. 2014), bee bread (Wang et al. 2018), and gut (Kang et al. 2021; Li and Gu 2022) of honeybees ( *Apis mellifera* ) and have been characterized in terms of taxonomy. Although no specific isolation sources were mentioned in these studies, this is likely the first time that lactic acid bacteria have been isolated from queen honeybees, which use RJ as their primary nutrition source. It is reasonable to suggest that some of the isolated lactic acid bacteria exhibiting the conversion activity of 10H2DA come from queen honeybees rather than worker honeybees. To our knowledge, the gut microbial communities of larvae and worker honeybees have been analyzed and characterized (Kim et al. 2023; Yang et al. 2024), but not those of queen honeybees. Although queen honeybee samples are rare and difficult to collect and analyze, comparing the composition of the 10H2DA‐converting lactic acid bacterium *L. panisapium* in the microbiota of worker and queen bees may highlight the importance of this bacterium.

The recombinant NADH‐dependent 2‐enoate reductase showed a narrow substrate specificity toward aromatic carboxylic acids, such as cinnamic acid and p‐coumaric acid, but not toward aliphatic 2‐enoates, such as crotonic acid and 2,3‐dimethylacrylic acid (Mordaka et al. 2018). The phylogenetic tree analysis indicated that the 2‐enoate reductase from *C. sporogenes* forms a cluster with various carbon–carbon double bond reductases from several Clostridium species but do not include the lactic acid bacteria group (Mordaka et al. 2018). 2‐Enoate reductases are catalytically active under microaerophilic fermentation conditions (Sun et al. 2016). Although the mechanism of conversion of 10H2DA to 10HDAA by *L. panisapium* M1 and its catalytic enzyme has not yet been revealed, *L. panisapium* M1 presumably has a different type of 2‐enoate reductase for 10H2DA. Further research is needed to establish an enzyme assay and to detect the reductase activity in cell‐free extracts from strain M1.

In cellular systems, the differentiation‐promoting effects on M cells and the activation of macrophage phagocytosis were observed, and in human tests, salivary IgA secretion rates increased (Figures 2, 3, 4, 5, 6). M cells, found in the intestinal epithelium, induce an immune response by taking up antigens and passing them on to immune cells. The number of M cells decreases with age in mice (Kobayashi et al. 2013). fRJ was more effective than eRJ in promoting M cell differentiation and activating macrophage phagocytosis (Figures 3, 4, 5). This differentiation‐promoting effect may reflect the conversion of 10H2DA to 10HDAA, which is presumably the active component. Additionally, RJ contains other fatty acids with double bonds, though not as abundant as 10H2DA; strain M1 may also contribute to the reduction of these fatty acids. Further research is needed to explore the M cell differentiation‐promoting effects of fatty acids other than 10HDAA.

Macrophages are leukocytes that phagocytose dead cells, their fragments, and external antigens. In this study, macrophage phagocytosis was activated more significantly by fRJ than by pRJ at 0.25 mg/mL, suggesting that 10HDAA and strain M1, characteristic components of fRJ, are active components. The activation of lactic acid bacteria, particularly strain M1, may contribute significantly to this effect.

IgA is an antibody found on the mucosal surfaces of the eyes, nose, throat, and intestines, preventing the entry of pathogens and viruses. Decreased IgA levels increase the incidence of upper respiratory tract infections (Neville et al. 2008). Since fRJ acts on M cells, which decline with age, and consequently stimulate salivary IgA secretion, it is thought to compensate for reduced immunity. In a previous study on cynomolgus monkeys, feeding the monkeys a human‐equivalent dose of 10HDAA (20 mg/day), similar to the present study, resulted in a 2–4‐fold increase in the expression of various antigen‐specific IgAs, aligning with increased GP2 expression (Isayama et al. 2020). These results are consistent with the findings of the current study.

The possibility of macrophage activation and enhanced IgA production has also been suggested for tempeh fermented from soya (Hosaka et al. 2021; Soka et al. 2015). Moreover, 10HDAA, as well as other fermentation products, may contribute to the various activities, and further verification is required.

The primary limitation to generalizing these results is that the differentiation‐promoting effect of 10HDAA on M cells has only been assessed in cell‐based evaluation systems. In this study, cell assays were performed using previously published methods (Isayama et al. 2020), and subsequent animal studies in the existing literature have confirmed M cell differentiation‐promoting effects in vivo. This suggests that fRJ also has M cell differentiation‐promoting effects in vivo, but this needs to be tested in animals in future research. Future work should also investigate the impact of promoting M cell differentiation on gut immunity.

## 5 Conclusion

The low level of 10HDAA in RJ was a problem, but by using strain M1 derived from the queen bee, it was possible to produce fRJ with a fivefold increase in 10HDAA over raw RJ. The fRJ was more effective at promoting M cell differentiation than pRJ, which was simply RJ treated with protease. Preliminary tests also showed that fRJ has the ability to activate macrophage phagocytosis and increase salivary IgA secretion rates, suggesting the possibility of using fRJ as an immunostimulatory food.

## Author Contributions

**Hayate Itatani:** conceptualization (equal), investigation (equal), project administration (equal), writing – original draft (equal). **Ayanori Yamaki:** conceptualization (equal), writing – review and editing (equal). **Kaori Konishi:** investigation (equal). **Hideto Okamoto:** investigation (equal). **Nobuaki Okumura:** supervision (equal), writing – review and editing (equal). **Norihiro Shigematsu:** supervision (equal). **Shogo Misumi:** investigation (equal), writing – review and editing (equal). **Shinji Takenaka:** investigation (equal), writing – review and editing (equal).

## Ethics Statement

The study was conducted with the approval of the Ethical Review Board of Yamada Bee Company Inc., and in accordance with the Declaration of Helsinki.

## Conflicts of Interest

The authors declare no conflicts of interest.

## Supporting information


Data S1.
**Figure S1**. HPLC separation and determination of 10‐hydroxy‐2‐decenoic acid (10H2DA) and 10‐hydroxydecanoic acid (10HDAA) in the cultural supernatants and raw royal jelly (RJ) and protease‐treated RJ. The isolate, strain M1, was anaerobically cultivated (b–e), as described in the main text. In addition, bioconversions of 10H2DA to 10HDAA were compared under aerobic and anaerobic conditions (f and g). (a) Standards, 10H2DA (retention time [min], 10.5), and 10HDAA (13.5); (b) control (without cultivation); (c) cultural supernatant in strain M1; (d) cultural supernatant in raw RJ broth; and (e) cultural supernatant in enzyme‐treated RJ broth. (f) Aerobic conditions and (g) anaerobic conditions.
**Figure S2**. IgA secretion rate (μg/min) in 12 subjects.
**Table S1**. Salivary immunoglobulin (Ig) A levels and salivary secretion rate.

## Data Availability

The datasets generated and/or analyzed during the current study are available in the repository at https://www.ncbi.nlm.nih.gov/nuccore/OR415255 (GenBank: OR415255.1). All data supporting this study are included in the published article or provided as Supporting Information.
