# Supplementary material for: Fermented Royal Jelly Enriched With 10‐Hydroxydecanoic Acid and Its Potential for Enhancing Mucosal Immunity
Source: Food Sci Nutr. 2025 Feb 18;13(2):e70041. doi: 10.1002/fsn3.70041 (PMC11833300; doi:10.1002/fsn3.70041)
Supplement: Supplementary file 1 — Data S1. Figure S1. HPLC separation and determination of 10‐hydroxy‐2‐decenoic acid (10H2DA) and 10‐hydroxydecanoic acid (10HDAA) in the cultural supernatants and raw royal jelly (RJ) and protease‐treated RJ. The isolate, strain M1, was anaerobically cultivated (b–e), as described in the main text. In addition, bioconversions of 10H2DA to 10HDAA were compared under aerobic and anaerobic conditions (f and g). (a) Standards, 10H2DA (retention time [min], 10.5), and 10HDAA (13.5); (b) control (without cultivation); (c) cultural supernatant in strain M1; (d) cultural supernatant in raw RJ broth; and (e) cultural supernatant in enzyme‐treated RJ broth. (f) Aerobic conditions and (g) anaerobic conditions. Figure S2. IgA secretion rate (μg/min) in 12 subjects. Table S1. Salivary immunoglobulin (Ig) A levels and salivary secretion rate. [file FSN3-13-e70041-s001.docx]

**
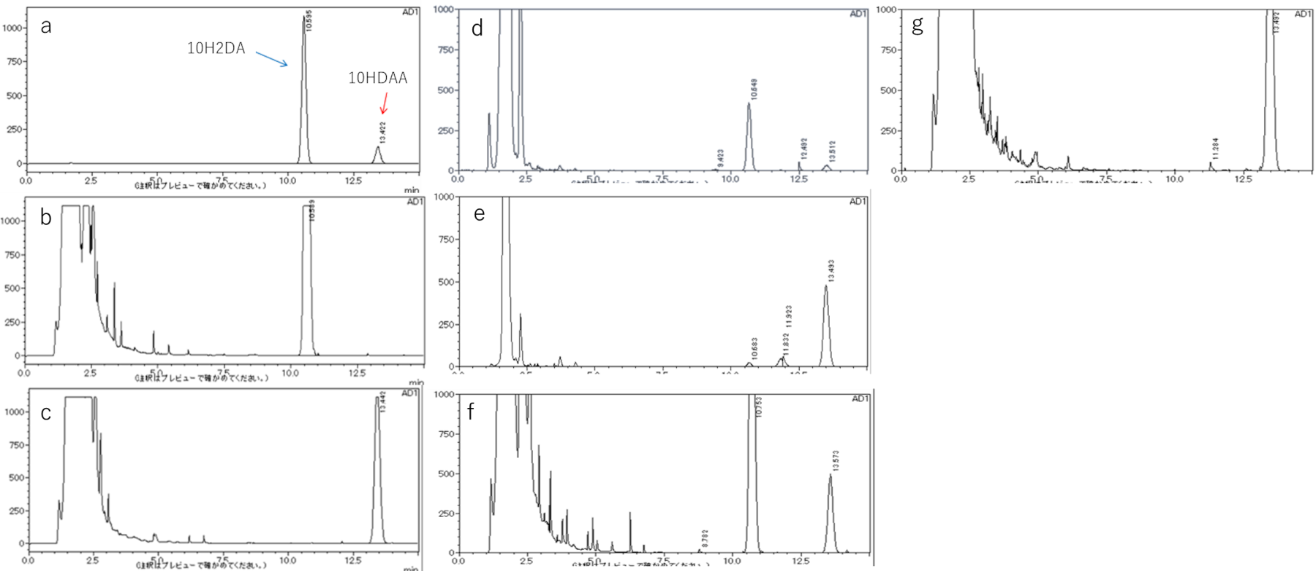
Supporting Information**

**Figure A1.** HPLC separation and determination of 10-hydroxy-2-decenoic acid (10H2DA) and 10-hydroxydecanoic acid (10HDAA) in the cultural supernatants and raw royal jelly (RJ) and protease-treated RJ. The isolate, strain M1, was anaerobically cultivated (b-e), as described in the main text. In addition, bioconversions of 10H2DA to 10HDAA were compared under aerobic and anaerobic conditions (f and g). (a) Standards, 10H2DA (retention time [min], 10.5), and 10HDAA (13.5); (b) control (without cultivation); (c) cultural supernatant in strain M1; (d) cultural supernatant in raw RJ broth; and (e) cultural supernatant in enzyme-treated RJ broth. (f) Aerobic conditions and (g) anaerobic conditions.


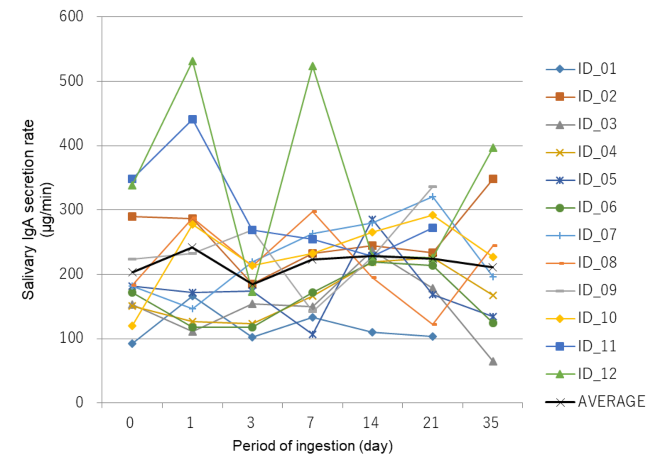


**Figure A2.** IgA secretion rate (μg/min) in 12 subjects.

**Table A1.** Salivary immunoglobulin (Ig) A levels and salivary secretion rate.

| Assessment items | Pre-ingestion | Period of ingestion | | | |
| --- | --- | --- | --- | --- | --- |
|  |  | 1 day | 2 weeks | 4 weeks | 8 weeks |
| **Salivary IgA secretion rate**  **(µg/min)** | **201.140 ± 24.711** | **189.654 ± 20.743** | **193.952 ± 23.649** | **213.722 ± 29.211** | **237.591 ± 30.019*** |
| **Salivary IgA concentration**  **(µg/mL)** | **584.544 ± 58.535** | **542.567 ± 41.040** | **516.148 ± 28.152** | **583.810 ± 41.482** | **644.681 ± 61.173** |
| **Salivary secretion rate**  **(mL/min)** | **0.395 ± 0.057** | **0.368 ± 0.032** | **0.393 ± 0.046** | **0.374 ± 0.043** | **0.409 ± 0.051** |

Comparison with pre-ingestion; *p < 0.05 (paired t-test); mean ± SD.
